# Knowledge, attitudes, and practices of exercise rehabilitation in patients following percutaneous coronary intervention

**DOI:** 10.3389/fcvm.2025.1706354

**Published:** 2026-01-20

**Authors:** Yanfu Wang, Yan Zhang, Wei Li, Yiming Men, Xinyu Ren, Chong Wang, Qifeng Jin

**Affiliations:** 1 Department of Cardiovascular Medicine, Aviation General Hospital, Beijing, China; 2 Department of Rehabilitation Medicine, Beijing Chest Hospital, Beijing, China; 3 Department of Heart Center, Beijing Chest Hospital, Beijing, China; 4 Department of Cardiology, Affiliated Hospital of Jining Medical College, Jining, Shandong, China

**Keywords:** attitudes, exercise rehabilitation, knowledge, percutaneous coronary intervention, practices

## Abstract

**Objective:**

To investigate the current knowledge, attitudes, and practices (KAP) status toward exercise rehabilitation in patients after percutaneous coronary intervention (PCI).

**Methods:**

This multi-center cross-sectional survey was conducted from March to May 2025 at three hospitals and enrolled patients who underwent PCI. It was conducted at Civil Aviation General Hospital in Beijing, the Affiliated Hospital of Jining Medical University in Shandong Province, and Tangta Hospital of Yuncheng County in Shanxi Province. A questionnaire was designed to collect participants’ demographic data and to assess KAP scores. The performance in each KAP dimension was categorized as poor (<60% of total score), moderate (60%–79%), or good (≥80%).

**Results:**

The final analysis included 346 valid questionnaires. The majority of participants demonstrated poor knowledge (167/346, 48.27%), whereas attitudes were predominantly moderate (176/346, 50.87%), and practices were largely good (168/346, 48.55%). The multivariable analysis showed that the knowledge scores (OR = 1.101, 95%CI: 1.024–1.185, *P* = 0.009), attitude scores (OR = 1.239, 95%CI: 1.134–1.355, *P* < 0.001), first PCI (OR = 2.354, 95%CI: 1.158–4.782, *P* = 0.018), and <1 month since the last PCI (OR = 2.400, 95%CI: 1.095–5.261, *P* = 0.029) were independently associated with proactive practice. The results of Structural Equation Modeling showed that knowledge directly influenced attitude (*β* = 0.518, *P* = 0.012) and practice (*β* = 0.432, *P* = 0.012). Attitude directly influenced practice (*β* = 0.444, *P* = 0.006). Knowledge indirectly influenced practice through its impact on attitude (*β* = 0.230, *P* = 0.008).

**Conclusion:**

The findings suggest that patients who underwent PCI had poor knowledge but moderate attitudes and good practices toward exercise rehabilitation. Patients with higher knowledge and attitude scores were more likely to report better practice scores, indicating associations rather than causal relationships. Future studies should explore targeted interventions and more rigorous research designs to better understand and potentially enhance KAP related to exercise rehabilitation after PCI.

## Introduction

In 2022, an estimated 19.8 million people died from cardiovascular diseases (CVDs), accounting for 32% of all global deaths. Of these, coronary heart disease (CHD) was the single largest contributor, responsible for around 9 million deaths per year, representing nearly 1 in 7 deaths worldwide (1, 2). In China specifically, cardiovascular diseases remain the leading cause of death, accounting for more than 40% of total mortality, with coronary heart disease contributing substantially to this burden according to recent national reports (3). Although the exact incidence is not available, the prevalence of CHD ranges in the hundreds of millions worldwide (1). CHD is primarily caused by the development of atherosclerotic plaques that progressively narrow the coronary artery lumen, thereby decreasing the nutrient and oxygen supply to the heart muscle (4–6). Atherosclerosis plaque rupture is an acute event that precipitates the formation of a thrombus, leading to ischemia of a part of the heart muscle (7).

Percutaneous coronary intervention (PCI) is an established technique that effectively alleviates vascular stenosis in coronary arteries by mechanically dilating the narrowed lumen and restoring blood flow. PCI achieves this by using balloon angioplasty and often deploying stents at sites of atherosclerotic narrowing (8, 9). While PCI can effectively manage the acute event related to vascular stenosis (8, 9), post-PCI exercise rehabilitation is strongly supported by robust evidence as a critical intervention to improve cardiac function, prevent restenosis, and enhance quality of life in patients following PCI (10–12). Self-management plays an essential and evidence-based role in exercise rehabilitation after PCI, significantly contributing to improved clinical outcomes, sustained exercise adherence, and enhanced quality of life. After the initial supervised phase, self-management empowers patients to continue structured exercise (often community- or home-based), which has been shown to maintain and even enhance improvements in cardiac function (e.g., left ventricular ejection fraction), physical capacity, and reduce the risk of major adverse cardiac events (13). Studies have shown that patients with higher self-management ability exhibit significantly better long-term outcomes, including notably reduced risks of restenosis, unplanned readmissions, and other adverse cardiovascular events, although the magnitude of these reductions varies across studies (14, 15). Self-management enables adjustment of exercise prescriptions based on patient feedback, symptoms, and preferences, allowing tailored interventions under remote or periodic supervision (13, 15). Still, self-management requires proper knowledge and attitudes. Interventions have been shown to effectively improve patient knowledge and foster more positive attitudes toward rehabilitation, which in turn contribute to better adherence and clinical outcomes (16–18). However, determining their level is necessary to design effective interventions.

Knowledge, Attitude, and Practice (KAP) studies are a widely used research methodology in public health, clinical medicine, and social sciences to systematically assess what a specific population knows, how they feel, and what they do regarding a particular topic, intervention, or health behavior (19, 20). KAP studies allow the identification of knowledge gaps, misconceptions, and misunderstandings that can hamper the optimal performance of a topic in a specific population (19, 20). Existing KAP research on patients after PCI has primarily focused on cardiac rehabilitation, postoperative self-management, or health education (21–24). These studies were conducted in various countries, including China, and used the KAP framework to evaluate patients’ understanding, attitudes, and engagement in lifestyle modification or rehabilitation programs. Notably, a recent KAP study conducted in China applied the KAP model to patients after coronary artery stenting, demonstrating its relevance and timeliness in the Chinese clinical context (21). However, few studies have specifically applied the KAP framework to exercise rehabilitation after PCI, highlighting the need addressed by the present study. Taken together, these global and national patterns highlight a clear gap in understanding exercise rehabilitation among PCI patients, which directly informs the objective of the present study. Based on previous findings, we hypothesized that higher levels of knowledge and more positive attitudes toward exercise rehabilitation would be associated with better rehabilitation practices among patients after PCI. Therefore, the present study aimed to assess the KAP status toward exercise rehabilitation among Chinese patients after PCI. Identifying gaps in patients’ knowledge, attitudes, and practices can provide evidence to guide the development of targeted intervention strategies, enhance patient education programs, and inform policy initiatives aimed at strengthening cardiovascular rehabilitation services.

## Methods

### Study design and participants

This multi-center cross-sectional survey was conducted from March to May 2025 at three hospitals (Aviation General Hospital in Beijing, the Affiliated Hospital of Jining Medical University in Shandong Province, and Tangta Hospital of Yuncheng County in Shanxi Province) and enrolled adult patients who underwent PCI. To maximize representativeness, all adult patients who underwent PCI at the three participating hospitals during the study period were consecutively included as the study population. The age range of enrolled adult patients was 18 years and older. The study protocol was approved by the ethics committee of Aviation General Hospital (HK-X-2025-01). Informed consent was obtained from all participants. The exclusion criteria were 1) physical mobility limitations, 2) patients who had undergone coronary artery bypass grafting (CABG), or 3) patients with other severe comorbidities (e.g., advanced-stage cancer, severe cardiopulmonary diseases).

### Questionnaire development

The investigators designed the questionnaire with reference to the literature (21–24) and clinical guidelines, including the *Expert Consensus on Postoperative Exercise Rehabilitation after Percutaneous Coronary Intervention* (25). After initial drafting, experts in psychology, cardiac rehabilitation, and chronic disease management provided feedback across three dimensions (psychological support, cardiac rehabilitation protocols, and long-term disease management) to ensure content validity. The key recommendations included strengthening psychological interventions for post-PCI patients, clarifying exercise rehabilitation protocols, and enhancing awareness of comprehensive long-term management. Revisions were made accordingly. A pilot study (*n* = 43) was conducted to assess reliability. The overall Cronbach's α coefficient for the KAP questionnaire was 0.966, with subscale values of 0.978 (Knowledge), 0.879 (Attitudes), and 0.957 (Practices), indicating excellent internal consistency. The pilot participants were also asked to indicate any statements that were unclear or difficult to understand, ensuring face validity. Construct validity of the questionnaire was further evaluated using confirmatory factor analysis (CFA). CFA was performed to examine whether the hypothesized three-factor structure (Knowledge, Attitudes, and Practices) adequately reflected the underlying constructs. The model demonstrated good fit to the data, with all key fit indices meeting recommended criteria (Supplementary Figure S1; Table S1). These results support the structural validity of the instrument.

The final questionnaire was in the Chinese language and comprised four sections: 1) demographic data (age, gender, education level, monthly household income per capita, height, weight, marital status, first-time PCI status, comorbidities, living arrangement), 2) knowledge dimension, 3) attitude dimension, and 4) practice dimension. The Body Mass Index (BMI) is calculated as the weight in kilograms divided by the square of the height in meters, with classifications of <18.5 kg/m^2^ as underweight, 18.5–23.9 kg/m^2^ as normal weight, 24–27.9 kg/m^2^ as overweight, and ≥28 kg/m^2^ as obesity(26). The comorbidities assessed in this study included the presence of one or more of the following conditions: diabetes mellitus, hypertension, hyperlipidemia, and heart failure. The knowledge section contained 11 items scored as “very familiar” = 2, “somewhat familiar” = 1, and “not familiar” = 0, yielding a total score range of 0–22. The attitude section consisted of 10 items, measured on a 5-point Likert scale (“strongly agree” = 5 to “strongly disagree” = 1), with total scores ranging from 10 to 50. Negative items were reverse-scored to ensure that higher total attitude scores consistently reflected more positive attitudes toward post-PCI exercise rehabilitation. This approach minimizes response bias and allows for uniform interpretation across all items. The practice section consisted of nine items scored from “never” = 1 to “always” = 5, resulting in a score range of 9–45. According to Bloom's cut-off criteria, the performance in each KAP dimension was categorized as poor (<60% of total score), moderate (60%–79%), or good (≥80%) (27, 28).

### Questionnaire distribution and quality control

This study employed convenience sampling with a hybrid online-offline distribution to reach a maximum of patients. QR codes were displayed in outpatient clinics and inpatient wards to recruit eligible participants. Trained research assistants addressed participant queries without influencing responses. The median time for participants to complete the questionnaire was 267 s (interquartile range: 191–403 s). To ensure data quality, four validity screening criteria were implemented. 1) A trap question was included: “K12. For this question, please select ‘c. Not familiar’”. Questionnaires with K12 = a or K12 = b were deemed invalid because the respondents were considered not to have read carefully or to have answered randomly. 2) Questionnaires completed in <60 s were excluded, as this duration was considered insufficient to ensure adequate comprehension and thoughtful responses. 3) Questionnaires with obvious data anomalies (e.g., age 150 years) were removed to minimize systematic errors and aberrant data affecting statistical analysis. 4) Repetitive response patterns (e.g., selecting the first option for all items) were excluded as they hinted at non-genuine participation.

### Statistical analysis

Statistical analysis was performed using SPSS 26.0 (IBM, Armonk, NY, USA) and AMOS 24.0 (IBM, Armonk, NY, USA). The continuous data were expressed as means ± standard deviations (SD). The continuous data were tested for normal distribution using the Kolmogorov–Smirnov test. Normally distributed data were analyzed using Student's *t*-test (two groups) or ANOVA (normal distribution with homogeneity of variance, three or more groups). Non-normal data were analyzed using the Mann–Whitney *U*-test (two groups) or the Kruskal–Wallis H-test (three or more groups). Spearman's correlation analyzed the correlations between KAP dimensions. Logistic regression was used to determine the factors associated with proactive practice. The variables with *P* < 0.05 in the univariate analyses were included in the multivariable model. Structural equation modeling (SEM) was employed to investigate the mediating effect of attitudes on the relationship between knowledge and practice. The SEM fit was evaluated using minimum discrepancy function by degrees of freedom divided (CMIN/DF) (<5 is good), root mean square error of approximation (RMSEA) (<0.08 is good), incremental fit index (IFI) (>0.08 is good), Tucker–Lewis index (TLI) (>0.8 is good), and comparative fit index (CFI) (>0.8 is good). Two-tailed *P*-values <0.05 were considered statistically significant.

## Results

### Characteristics of the participants

Among 419 returned questionnaires, 73 were excluded: no consent (*n* = 3), completion time <60 s (*n* = 1), data anomalies (*n* = 5), failed trap question (*n* = 58), and uniform KAP responses (*n* = 6). Hence, the final analysis included 346 valid questionnaires, yielding an effective response rate of 82.58%.

The participant characteristics are summarized in Table 1. Most were male (65.03%), aged >45 years (66.47%), and had a BMI ≥24 kg/m^2^ (53.18%). The majority lived in urban areas (75.43%), were married (92.49%), and had at least one comorbidity (86.99%). Most were undergoing their first PCI (76.59%), and 40.46% had the procedure more than six months before the survey. Only 7.8% lived alone, and 66.8% had received education on postoperative exercise rehabilitation before. The majority of participants demonstrated poor knowledge (167/346, 48.27%), whereas attitudes were predominantly moderate (176/346, 50.87%), and practices were largely good (168/346, 48.55%) (Supplementary Table S2).

**Table 1:** Characteristics of the participants.

Characteristics	*n* (%)	Knowledge (mean ± SD)	*P*-value	Attitude (mean ± SD)	*P*-value	Practice (mean ± SD)	*P*-value
*N* = 346							
Total score		12.96 ± 5.63		38.99 ± 4.72		34.38 ± 6.15	
Age (years)			<0.001		<0.001		<0.001
≤45	116 (33.53%)	14.15 ± 5.02		40.41 ± 4.25		36.28 ± 5.29	
45–60	132 (38.15%)	13.48 ± 5.52		39.20 ± 4.13		34.29 ± 6.32	
>60	98 (28.32%)	10.85 ± 5.92		37.02 ± 5.32		32.26 ± 6.21	
Gender			0.995		0.067		0.840
Male	225 (65.03%)	12.94 ± 5.84		39.34 ± 4.58		34.31 ± 6.21	
Female	121 (34.97%)	12.99 ± 5.22		38.32 ± 4.92		34.51 ± 6.07	
BMI (kg/m^2^)			0.436		0.793		0.869
Underweight (<18.5)	10 (2.89%)	12.50 ± 5.58		40.10 ± 6.40		35.50 ± 6.33	
Normal (18.5–23.9)	152 (43.93%)	13.20 ± 5.60		39.06 ± 4.81		34.33 ± 6.19	
Overweight or obesity (≥24)	184 (53.18%)	12.79 ± 5.67		38.86 ± 4.56		34.36 ± 6.14	
Place of residence			0.001		<0.001		<0.001
Rural	85 (24.57%)	11.38 ± 6.04		37.21 ± 4.76		31.92 ± 6.21	
Urban	261 (75.43%)	13.48 ± 5.39		39.56 ± 4.57		35.18 ± 5.93	
Education level			<0.001		<0.001		<0.001
Junior high school or below	83 (23.99%)	9.90 ± 5.36		36.06 ± 4.59		30.60 ± 5.77	
High school/Technical secondary school	57 (16.47%)	14.21 ± 5.50		40.04 ± 4.27		36.02 ± 6.07	
Associate degree	72 (20.81%)	13.22 ± 5.60		39.19 ± 4.55		34.47 ± 6.35	
Bachelor's degree	111 (32.08%)	14.32 ± 4.97		39.77 ± 4.06		36.03 ± 5.26	
Master's degree or above	23 (6.65%)	13.52 ± 6.19		42.48 ± 4.87		35.74 ± 5.59	
Monthly income per capita (CNY)			<0.001		<0.001		<0.001
<2,000	29 (8.38%)	10.66 ± 6.20		36.31 ± 5.06		32.10 ± 6.48	
2,000–4,999	92 (26.59%)	10.41 ± 5.69		36.83 ± 4.50		31.75 ± 6.31	
5,000–9,999	112 (32.37%)	13.96 ± 5.17		40.20 ± 4.06		35.60 ± 6.12	
10,000–20,000	76 (21.97%)	14.37 ± 4.51		39.99 ± 4.23		36.14 ± 3.90	
>20,000	37 (10.69%)	15.19 ± 5.81		40.73 ± 5.15		35.41 ± 6.95	
Marital status			0.095		0.538		0.147
Married	320 (92.49%)	13.14 ± 5.41		38.93 ± 4.54		34.28 ± 5.89	
Other	26 (7.51%)	10.77 ± 7.61		39.62 ± 6.62		35.69 ± 8.86	
First PCI			<0.001		<0.001		<0.001
Yes	265 (76.59%)	13.59 ± 5.41		39.56 ± 4.64		35.11 ± 5.91	
No	81 (23.41%)	10.89 ± 5.86		37.10 ± 4.48		31.99 ± 6.36	
Time since the most recent PCI procedure			<0.001		0.013		<0.001
Within the past 1 month	102 (29.48%)	11.05 ± 5.23		37.67 ± 4.81		33.27 ± 5.57	
1–3 months	63 (18.21%)	14.79 ± 4.40		39.32 ± 4.19		36.84 ± 4.58	
3–6 months	41 (11.85%)	14.32 ± 5.19		39.85 ± 5.13		34.78 ± 5.98	
6 months and more	140 (40.46%)	13.13 ± 6.12		39.54 ± 4.60		33.96 ± 6.92	
Other comorbidities			0.635		0.644		0.831
Yes	301 (86.99%)	12.93 ± 5.66		38.97 ± 4.80		34.41 ± 6.17	
No	45 (13.01%)	13.13 ± 5.47		39.07 ± 4.18		34.18 ± 6.11	
Living alone			0.020		0.006		<0.001
Yes	27 (7.80%)	10.33 ± 6.37		36.70 ± 5.41		29.89 ± 7.32	
No	319 (92.20%）	13.18 ± 5.51		39.18 ± 4.61		34.76 ± 5.90	
Education regarding postoperative exercise rehabilitation			<0.001		<0.001		<0.001
Yes	231 (66.76%)	14.85 ± 4.63		39.81 ± 4.43		35.81 ± 5.45	
No	115 (33.24%)	9.16 ± 5.55		37.34 ± 4.85		31.51 ± 6.50	

BMI, body mass index.

### Knowledge

The mean knowledge score among all participants was 12.96 ± 5.63 (on a possible maximum of 22; 58.91%), indicating poor knowledge. Knowledge scores were significantly associated with age, residence, educational attainment, monthly income, PCI history, time since the most recent PCI, living arrangement, and receipt of postoperative exercise rehabilitation education (all *P* < 0.05) (Table 1). Participants’ familiarity and understanding of post-PCI exercise rehabilitation varied across specific knowledge items. Overall, only a minority of participants reported being “very familiar” with most domains, while the majority had at least “heard of” key principles, and a small proportion remained “not sure” (Supplementary Table S3).

### Attitude

The mean attitude score for all participants was 38.99 ± 4.72 (out of a maximum of 50; 77.98%), indicating a moderate attitude. Age, place of residence, education level, monthly income, first PCI status, time since the most recent PCI, living arrangement, and participation in postoperative exercise rehabilitation education were all significantly associated with attitude scores (all *P* < 0.05) (Table 1). Participants generally expressed positive attitudes toward exercise rehabilitation following PCI. Among the attitude items, participants showed the most positive attitudes toward post-PCI exercise rehabilitation in items A1 (“Patients should participate in exercise rehabilitation”), A2 (“Exercise is highly beneficial for cardiac health”), and A7 (“Willingness to cooperate with the prescribed exercise plan”), with 87.57%, 88.15%, and 89.02% of respondents selecting “strongly agree” or “agree,” respectively. Conversely, the least positive attitudes were observed in items A3 (“Worry that exercise might trigger cardiac recurrence”) and A10 (“Concern about being unable to adhere to the plan”), with 40.17% and 36.12% selecting “strongly agree” or “agree,” indicating notable concerns regarding exercise-related risks and adherence (Supplementary Table S4).

### Practice

The average practice score among all participants was 34.38 ± 6.15 (out of a maximum of 45; 76.40%), indicating moderate practice proficiency. Place of residence, education level, monthly income, first PCI status, time since the most recent PCI, living arrangement, and participation in postoperative exercise rehabilitation education were all significantly associated with practice scores (all *P* < 0.001) (Table 1). Participants reported the highest adherence for item 6 (“I avoid high-intensity exercise”), with 81.79% selecting “always” or “often.” High adherence was also observed for items 2 (“Undergo regular cardiac health check-ups,” 67.34%) and 7 (“Adjust exercise intensity based on health status,” 75.43%). Conversely, lower adherence was noted for item 9 (“Regularly discuss exercise effectiveness with doctor and adjust plan accordingly”), with only 50.86% reporting “always” or “often,” indicating room for improvement in active engagement with medical guidance (Supplementary Table S5).

### Multivariable analysis of practice

The multivariable analysis showed that the knowledge scores (OR = 1.101, 95%CI: 1.024–1.185, *P* = 0.009), attitude scores (OR = 1.239, 95%CI: 1.134–1.355, *P* < 0.001), first PCI (OR = 2.354, 95%CI: 1.158–4.782, *P* = 0.018), and <1 month since the last PCI (OR = 2.400, 95%CI: 1.095–5.261, *P* = 0.029) were independently associated with proactive practice (Table 2).

**Table 2:** Univariate and multivariable analysis of the practice dimension.

Characteristics	Univariate logistic regression		Multivariable logistic regression	
	OR (95%CI)	*P*-value	OR (95%CI)	*P*-value
Knowledge	1.232 (1.166–1.302)	<0.001	1.101 (1.024–1.185)	0.009
Attitude	1.339 (1.242–1.444)	<0.001	1.239 (1.134–1.355)	<0.001
Age (years)				
≤45	7.065 (3.651–13.673)	<0.001	1.537 (0.541–4.363)	0.419
45–60	3.014 (1.737–5.233)	<0.001	1.064 (0.481–2.354)	0.878
>60	ref		ref	
Gender				
Male	1.171 (0.726–1.888)	0.518		
Female	ref			
BMI (kg/m^2^)				
<18.5	0.969 (0.242–3.888)	0.965		
18.5–23.9	0.928 (0.581–1.482)	0.754		
≥24	ref			
Place of residence				
Rural	0.242 (0.144–0.406)	<0.001	0.618 (0.268–1.427)	0.259
Urban	ref		ref	
Education level				
Junior high school or below	0.166 (0.056–0.491)	0.001	1.066 (0.161–7.064)	0.947
High school/ Technical secondary school	0.712 (0.226–2.241)	0.561	1.761 (0.321–9.648)	0.515
Associate degree	0.899 (0.290–2.783)	0.853	3.108 (0.560–17.246)	0.195
Bachelor's degree	1.925 (0.617–6.007)	0.260	4.374 (0.862–22.198)	0.075
Master's degree or above	ref		ref	
Monthly income per capita				
<2,000	0.181 (0.058–0.564)	0.003	1.687 (0.294–9.686)	0.558
2,000–4,999	0.177 (0.068–0.466)	<0.001	0.702 (0.163–3.029)	0.636
5,000–9,999	0.609 (0.230–1.616)	0.320	0.481 (0.121–1.912)	0.298
10,000–20,000	1.645 (0.526–5.147)	0.392	1.640 (0.369–7.286)	0.515
>20,000	ref		ref	
Marital status				
Married	1.037 (0.436–2.467)	0.934		
Other	ref			
First PCI				
Yes	3.526 (2.095–5.933)	<0.001	2.354 (1.158–4.782)	0.018
No	ref		ref	
Time since the most recent PCI procedure				
Within the past 1 month	0.734 (0.431–1.251)	0.256	2.400 (1.095–5.261)	0.029
1–3 months	3.257 (1.431–7.410)	0.005	2.131 (0.770–5.902)	0.145
3–6 months	1.292 (0.594–2.809)	0.518	0.781 (0.252–2.419)	0.669
6 months and more	ref		ref	
Other comorbidities				
Yes	0.826 (0.408–1.671)	0.595		
No	ref			
Living alone				
Yes	0.310 (0.139–0.687)	0.004	0.350 (0.110–1.116)	0.076
No	ref		ref	
Education regarding postoperative exercise rehabilitation				
Yes	4.092 (2.513–6.665)	<0.001	1.948 (0.926–4.098)	0.079
No	ref		ref	

OR, odds ratio; CI, confidence interval; BMI, body mass index.

### Correlation analyses

The knowledge scores were correlated with the attitude (*r* = 0.531, *P* < 0.001) and practice (*r* = 0.606, *P* < 0.001) scores. The attitude scores were correlated with the practice scores (*r* = 0.596, *P* < 0.001) (Table 3).

**Table 3:** Correlation analysis.

Domains	Knowledge	Attitude	Practice
Knowledge	1		
Attitude	0.531 (*P* < 0.001)	1	
Practice	0.606 (*P* < 0.001)	0.596 (*P* < 0.001)	1

### SEM analysis

**Figure 1:**
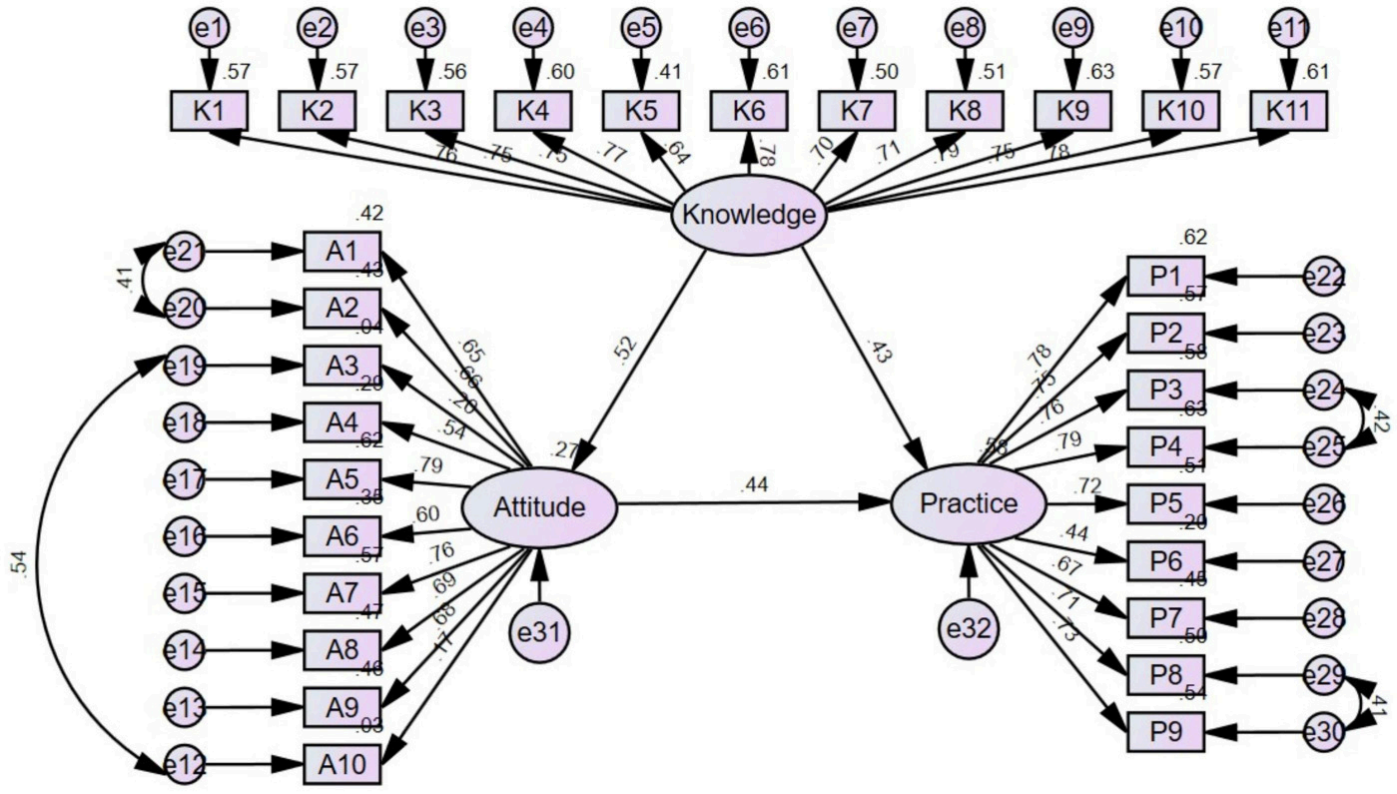
Structural equation model (SEM) depicting the relationships among the latent variables: knowledge, attitude, and practice. Ellipses represent latent variables (unobserved constructs), including overall knowledge, attitude, and practice. Rectangles represent observed variables (measured questionnaire items) that serve as indicators of the latent variables. Circles represent error terms or residuals associated with the observed variables. Single-headed arrows indicate hypothesized directional relationships (pathways) between latent variables and from latent variables to observed variables, with standardized path coefficients labeled on the arrows.

Supplementary Table S6 shows that all SEM fit indexes indicated a good model fit. Based on the SEM model (Figure 1), knowledge directly influenced attitude (*β* = 0.518, *P* = 0.012) and practice (*β* = 0.432, *P* = 0.012). Attitude directly influenced practice (*β* = 0.444, *P* = 0.006). Knowledge indirectly influenced practice through attitude (*β* = 0.230, *P* = 0.008) (Table 4).

**Table 4:** Direct and indirect relationships in the SEM model.

Model paths	Standardized total effects (95%CI)	*P*-value	Standardized direct effects (95%CI)	*P*-value	Standardized indirect effects (95%CI)	*P*-value
Knowledge → Attitude	0.518 (0.400–0.617)	0.012	0.518 (0.400–0.617)	0.012		
Knowledge → Practice	0.662 (0.549–0.740)	0.018	0.432 (0.287–0.586)	0.012	0.230 (0.136–0.323)	0.008
Attitude → Practice	0.444 (0.278–0.607)	0.006	0.444 (0.278–0.607)	0.006		

CI, confidence interval.

## Discussion

The results of this study suggested that patients who underwent PCI had poor knowledge (58.91% of the total possible score) but had a moderate attitude and practice toward exercise rehabilitation (77.98% and 76.40% of the total possible scores, respectively). Participants with moderate or good knowledge scores were more likely to report moderate or good practice scores, compared with those with poorer knowledge performance, further illustrating the associations observed in this cross-sectional study. Based on the gaps observed in the present study, interventions to improve the KAP toward exercise rehabilitation after PCI should be designed and tested.

The finding that more than half of post-PCI patients were at least familiar with fundamental concepts of exercise rehabilitation, yet a substantial proportion exhibited gaps in detailed or in-depth knowledge, as only a minority of participants reported being “very familiar” with most knowledge items, highlights a clear need for enhanced and targeted patient education in this population, which is supported by the available literature. Indeed, similar deficiencies in patient knowledge following PCI have been reported in both Chinese and international studies. For example, a recent KAP survey from China (21) found that although most patients undergoing coronary artery stenting recognized the importance of cardiac rehabilitation, their understanding of specific elements (such as exercise timing, risk management, and long-term adherence) was consistently lacking, with only a minority possessing comprehensive knowledge. It indicates a persistent gap between general awareness and in-depth understanding, which supports the present study. Education interventions have been shown to significantly improve knowledge; however, baseline assessments often reveal moderate to poor knowledge among post-PCI patients. For instance, in a multicenter trial, education level and prior exposure to cardiac rehabilitation were the strongest predictors of post-PCI knowledge improvement, emphasizing the necessity of structured, ongoing patient education to address these deficits (16). Guidelines and review articles also reinforce that patient education is a core component of successful cardiac rehabilitation, but report that detailed knowledge is often insufficient. Reports suggest the need for more individualized, culturally adapted educational programs to enhance both patient outcomes and engagement in exercise rehabilitation after PCI (17, 18, 29).

Overall, attitudes toward post-PCI exercise rehabilitation among this cohort were highly favorable, with strong agreement on its benefits, willingness to participate and adjust lifestyle, and the importance of professional and familial support. Nonetheless, a notable proportion of patients reported concerns about safety and long-term adherence, suggesting that targeted education and ongoing support may be beneficial in addressing these anxieties and promoting sustained engagement in rehabilitation activities. Studies from China and other countries routinely report that post-PCI patients appreciate the benefits of exercise rehabilitation, exhibiting a willingness to participate and adjust their lifestyle habits for improved cardiac health. Favorable attitudes are positively correlated with higher education, urban residence, and exposure to professional guidance. Most patients view professional support and family involvement as critical facilitators for engagement in rehabilitation activities (21, 30–32). Despite favorable beliefs, a substantial proportion of patients express anxiety about exercise-induced cardiac events, concerns about physical limitations, and doubts regarding their ability to sustain long-term adherence. Barriers frequently cited include fear of recurrence or worsening of disease and anxiety about exercising safely, physical and emotional limitations (such as fatigue, depression, and uncertainty about what constitutes safe exercise), and lack of confidence in independent exercise planning/monitoring, especially without direct oversight or peer support (30, 33). Previous studies emphasized that these anxieties can lead to suboptimal adherence, with overall participation in cardiac rehabilitation programs remaining moderate to low among post-PCI patients. Adherence is further affected by social support deficits, lower education, and limited access to rehabilitation resources (23, 30, 33). The available literature strongly suggests that targeted, structured education (delivered by healthcare professionals) and ongoing psychosocial support both reduce patient anxieties and enable better sustained engagement in exercise rehabilitation. Well-designed educational interventions build both knowledge and confidence, helping patients distinguish beneficial activity from harmful exertion, and foster motivation for long-term participation (30, 34). Ongoing psychological and family-based support is recommended to reinforce positive attitudes and minimize drop-out, particularly in populations with lower baseline knowledge or greater psychological vulnerability (30, 32, 34). Tailored programs considering individual psychosocial and demographic factors are consistently advocated to address the noted gaps (14, 30, 33).

While a substantial proportion reported frequent or consistent engagement in recommended exercise and self-management behaviors, including 81.79% who avoided high-intensity exercise, 75.43% who adjusted exercise intensity based on their health status, and 67.34% who underwent regular cardiac check-ups, others exhibited irregular or infrequent adherence, particularly in areas such as monitoring, physician consultation, and plan adjustment. These findings underscore the need for ongoing support and patient-centered interventions to promote optimal rehabilitation practice and long-term secondary prevention. Recent studies confirm that adherence rates to both phase I (in-hospital) and phase II (post-discharge) cardiac rehabilitation after PCI are modest, with only about 30%–35% of patients demonstrating consistently high adherence. Most patients fall into intermediate or low adherence categories, particularly outside formal rehabilitation settings. Factors contributing to suboptimal practice include limited knowledge, low self-efficacy, lack of social or family support, psychological barriers (fear of recurrence, anxiety), and poor access to tailored resources or ongoing interaction with healthcare providers (23, 35–37). The literature notes recurring deficiencies in self-monitoring (e.g., tracking heart rate, symptoms), proactive physician consultation upon noticing abnormal exercise responses, and regular review or adjustment of rehabilitation plans. Patients are less likely to maintain these behaviors over time without structured, ongoing contact with their care teams. Physicians’ active endorsement and structured referral to cardiac rehabilitation are key predictors of participation and compliance. Integration between hospital and outpatient care teams can facilitate better adherence and outcomes (23, 35, 36, 38, 39). Patient-centered interventions are increasingly recommended to address these challenges. Indeed, home-based or mobile health-enhanced cardiac rehabilitation yields markedly higher adherence rates, reflecting benefits from flexible, personalized support combined with frequent monitoring and feedback (14, 35). Case management and nurse-led interventions enhance both exercise adherence and follow-up, resulting in decreased readmissions and adverse cardiovascular events (14). Couple- or family-based interventions show promise in improving self-management and psychological well-being, leveraging the critical role of social support in adherence (32). Tailored education and counseling programs, specifically addressing knowledge gaps and psychological barriers, are vital. They foster increased self-efficacy and help patients overcome fears or misconceptions about safe exercise post-PCI (36).

Hence, as shown by the correlation, multivariable, and SEM analyses, interventions that could improve knowledge and attitude should result in improved practice of exercise rehabilitation after PCI. In this study, participants with more favorable knowledge and attitude scores tended to report better practice levels, highlighting the positive associations among the KAP dimensions without implying causality. Future studies should design and test such interventions, which could take different forms, such as reading materials, interactive websites, videos, and podcasts, for example. Future research should also consider integrating additional theoretical frameworks, such as the Health Belief Model, Theory of Planned Behavior, or Social Cognitive Theory, to more comprehensively explain the mechanisms linking knowledge, attitudes, and practices. Incorporating such frameworks may enhance study design, improve variable selection, and provide a stronger basis for interpreting behavioral pathways. To enhance practical applicability, future intervention programs should consider incorporating structured educational modules, standardized exercise guidance, individualized risk assessment, and periodic follow-up support delivered through hospital-based sessions, community programs, or digital platforms. Providing clear instructional content and incorporating patient feedback mechanisms may further improve feasibility and adherence.

Furthermore, the present study showed that knowledge and attitude were associated with socioeconomic status. A higher socioeconomic status is a well-known determinant of health literacy (40). The study also showed that patients not undergoing their first PCI or with PCI performed more than 1 month ago had poorer practice scores, highlighting that such patients should be targeted first by the intervention. Additionally, although socioeconomic and demographic variables such as education, residence, and income were included in the regression analyses, other important confounders, such as psychological status, comorbid depression, and access to rehabilitation resources, were not assessed or adjusted for, which may have influenced the observed KAP outcomes.

The present study had limitations. The patients were from three hospitals, limiting the sample size. The participants were also from a limited geographical area, which limited the generalizability of the results. Furthermore, because all participating hospitals were located in specific regions of China, the findings may not be fully generalizable to populations from other cultural, socioeconomic, or healthcare contexts. Furthermore, KAP studies have several methodological limitations that should be acknowledged. First, as questionnaire-based surveys, KAP studies may only capture a surface-level understanding of knowledge, attitudes, and practices, potentially overlooking important psychosocial variables such as perceived risks, social pressures, and personal motivations. The responses are often self-reported and may be subject to social desirability bias (41, 42), leading participants to overstate socially acceptable attitudes or practices rather than reflect true behaviors. Poorly designed survey instruments or ambiguous questions can compromise data validity and reliability, while inadequate consideration of cultural or contextual factors may lead to misinterpretation by respondents. Additionally, the cross-sectional design of most KAP studies precludes the ability to make causal inferences. It limits the ability to track changes over time or determine if improvements in knowledge and attitudes result in sustained behavioral change. A SEM analysis was performed to examine how KAP dimensions influenced each other, but such an analysis does not indicate causality (43–45). Finally, the lack of standardized sample size estimation procedures and potential selection bias may compromise the generalizability of the findings. Additionally, the use of convenience sampling and a hybrid online–offline recruitment approach may have introduced further selection bias, as no randomization or stratification procedures were applied to ensure representativeness. Moreover, the study relied solely on self-reported questionnaires, without any objective measures to validate actual exercise adherence or practice behaviors, which may have introduced reporting bias. Although the questionnaire demonstrated satisfactory psychometric performance—including high internal consistency, good content validity supported by expert review, and adequate construct validity confirmed through CFA—it remains a self-developed instrument. The tool has not yet been validated against external standardized questionnaires, which should be addressed in future research to further strengthen its measurement properties.

To strengthen future research in this field, studies should consider recruiting larger and more diverse populations, incorporating validated and objective measures of exercise behavior, employing longitudinal or interventional designs to better assess causal pathways, and integrating strong theoretical frameworks to more clearly explain how knowledge and attitudes translate into behavior change. In conclusion, Patients who underwent PCI demonstrated limited knowledge yet exhibited moderate attitudes and practices regarding exercise rehabilitation. Enhancing knowledge and attitudes is expected to lead to improved practice. In light of the gaps identified in this study, targeted interventions to strengthen KAP related to post-PCI exercise rehabilitation should be developed and systematically evaluated.

## Data Availability

The original contributions presented in the study are included in the article/Supplementary Material, further inquiries can be directed to the corresponding author.
