# Supplementary material for: Knowledge, attitudes, and practices of exercise rehabilitation in patients following percutaneous coronary intervention
Source: Front Cardiovasc Med. 2026 Jan 20;12:1706354. doi: 10.3389/fcvm.2025.1706354 (PMC12864515; doi:10.3389/fcvm.2025.1706354)
Supplement: Supplementary file 1 [file Datasheet1.docx]

**Table S1.** Model fit index of confirmatory factor analysis

| **Indicators** | **Reference** | **Actual** |
| --- | --- | --- |
| **CMIN/DF** | 1-3: Excellent, 3-5: Good | 2.705 |
| **RMSEA** | <0.08: Good | 0.070 |
| **IFI** | >0.8: Good | 0.881 |
| **TLI** | >0.8: Good | 0.870 |
| **CFI** | >0.8: Good | 0.880 |

**Table S2.** **Distribution of Knowledge, Attitude, and Practice Categories Among Participants**

|  | **Poor (<60%)** | **Moderate (60%-76%)** | **Good (≥80%)** |
| --- | --- | --- | --- |
| **Knowledge** | 167(48.27) | 57(16.47) | 122(35.26) |
| **Attitude** | 18(5.20) | 176(50.87) | 152(43.93) |
| **Practice** | 47(13.58) | 131(37.86) | 168(48.55) |

**Table S3.** Distribution of the knowledge responses

| **Knowledge items, n (%)** | **Very familiar** | **Somewhat familiar** | **Not familiar** |
| --- | --- | --- | --- |
| 1.Post-PCI exercise rehabilitation refers to a structured, progressive physical activity program guided by healthcare professionals to promote cardiac health and overall recovery. | 85 (24.57%) | 203 (58.67%) | 58 (16.76%) |
| 2.Post-PCI exercise rehabilitation positively impacts cardiac health. | 102 (29.48%) | 193 (55.78%) | 51 (14.74%) |
| 3.Regular evaluation of exercise effectiveness and adjustment of exercise plans are integral to post-PCI rehabilitation. | 95 (27.46%) | 189 (54.62%) | 62 (17.92%) |
| 4.Healthcare providers typically recommend initiating light exercise approximately one week after PCI. | 126 (36.42%) | 170 (49.13%) | 50 (14.45%) |
| 5.Suitable post-PCI exercises include low-intensity activities such as walking, swimming, and cycling. | 104 (30.06%) | 190 (54.91%) | 52 (15.03%) |
| 6.Post-PCI patients should adjust exercise intensity and frequency based on individual tolerance. | 116 (33.53%) | 186 (53.76%) | 44 (12.72%) |
| 7.Avoiding overexertion is critical during post-PCI exercise. | 149 (43.06%) | 167 (48.27%) | 30 (8.67%) |
| 8.Post-PCI patients should recognize potential cardiac warning signs (e.g., chest pain, shortness of breath) during exercise. | 121 (34.97%) | 182 (52.60%) | 43 (12.43%) |
| 9.Warm-up and cool-down activities reduce exercise-related risks after PCI. | 107 (30.92%) | 168 (48.55%) | 71 (20.52%) |
| 10.Long-term adherence to appropriate exercise reduces the risk of cardiac recurrence post-PCI. | 115 (33.24%) | 185 (53.47%) | 46 (13.29%) |
| 11.Post-PCI patients should consult healthcare providers for personalized exercise recommendations. | 121 (34.97%) | 169 (48.84%) | 56 (16.18%) |

**Table S4.** Distribution of the knowledge responses

| **Attitude items, n (%)** | **Strongly agree** | **Agree** | **Neutral** | **Disagree** | **Strongly disagree** |
| --- | --- | --- | --- | --- | --- |
| 1.I believe patients should participate in exercise rehabilitation after PCI. | 93 (26.88%) | 210 (60.69%) | 36 (10.40%) | 5 (1.45%) | 2 (0.58%) |
| 2.I think exercise is highly beneficial for improving cardiac health. | 120 (34.68%) | 185 (53.47%) | 35 (10.12%) | 4 (1.16%) | 2 (0.58%) |
| 3.I worry that exercise might trigger a cardiac recurrence. (N) | 20 (5.78%) | 119 (34.39%) | 109 (31.50%) | 84 (24.28%) | 14 (4.05%) |
| 4.I believe the benefits of postoperative exercise outweigh potential risks. | 60 (17.34%) | 209 (60.40%) | 59 (17.05%) | 14 (4.05%) | 4 (1.16%) |
| 5.I am willing to adjust my lifestyle habits to accommodate post-PCI exercise rehabilitation. | 118 (34.10%) | 182 (52.60%) | 42 (12.14%) | 4 (1.16%) | 0 |
| 6.I think exercise rehabilitation requires guidance and support from a professional rehabilitation team. | 104 (30.06%) | 188 (54.34%) | 47 (13.58%) | 5 (1.45%) | 2 (0.58%) |
| 7.I am willing to actively cooperate with the exercise rehabilitation plan prescribed by my doctor. | 132 (38.15%) | 176 (50.87%) | 36 (10.40%) | 2 (0.58%) | 0 |
| 8.I believe exercise rehabilitation can prolong life expectancy. | 108 (31.21%) | 190 (54.91%) | 41 (11.85%) | 7 (2.02%) | 0 |
| 9.I think post-PCI exercise rehabilitation requires collaborative efforts from family members. | 108 (31.21%) | 187 (54.05%) | 45 (13.01%) | 6 (1.73%) | 0 |
| 10.I worry about being unable to adhere to the treatment plan and giving up midway. (N) | 18 (5.20%) | 107 (30.92%) | 90 (26.01%) | 109 (31.50%) | 22 (6.36%) |

Note: N indicates a reverse-scored item.

**Table S5.** Distribution of the practice responses

| **Practice items, n (%)** | **Always** | **Often** | **Sometimes** | **Rarely** | **Never** |
| --- | --- | --- | --- | --- | --- |
| 1.Since my PCI, I have started exercise rehabilitation as recommended by my doctor. | 57 (16.47%) | 168 (48.55%) | 94 (27.17%) | 22 (6.36%) | 5 (1.45%) |
| 2.I undergo regular cardiac health check-ups. | 95 (27.46%) | 138 (39.88%) | 90 (26.01%) | 19 (5.49%) | 4 (1.16%) |
| 3.I am able to find a suitable time for exercise in my daily routine. | 68 (19.65%) | 154 (44.51%) | 109 (31.50%) | 13 (3.76%) | 2 (0.58%) |
| 4.I follow my planned exercise regimen. | 60 (17.34%) | 158 (45.66%) | 98 (28.32%) | 28 (8.09%) | 2 (0.58%) |
| 5.I monitor my physical responses (e.g., heart rate, breathing) during or after exercise. | 90 (26.01%) | 128 (36.99%) | 100 (28.90%) | 24 (6.94%) | 4 (1.16%) |
| 6.I avoid high-intensity exercise. | 171 (49.42%) | 112 (32.37%) | 48 (13.87%) | 11 (3.18%) | 4 (1.16%) |
| 7.I adjust exercise intensity based on my health status. | 99 (28.61%) | 162 (46.82%) | 67 (19.36%) | 16 (4.62%) | 2 (0.58%) |
| 8.I consult my doctor promptly if I notice any abnormal symptoms during exercise. | 88 (25.43%) | 126 (36.42%) | 95 (27.46%) | 31 (8.96%) | 6 (1.73%) |
| 9.I regularly discuss the effectiveness of my exercise rehabilitation with my doctor and adjust the plan accordingly. | 67 (19.36%) | 109 (31.50%) | 112 (32.37%) | 47 (13.58%) | 11 (3.18%) |

**Table S6.** SEM model fit results

| Model one | Ref. | Measured results |
| --- | --- | --- |
| CMIN/DF | 1-3 excellent, 3-5 good | 2.024 |
| RMSEA | <0.08 good | 0.054 |
| IFI | >0.8 good | 0.929 |
| TLI | >0.8 good | 0.922 |
| CFI | >0.8 good | 0.929 |

CMIN/DF: minimum discrepancy function by degrees of freedom divided; RMSEA: root mean square error of approximation; IFI: incremental fit index; TLI: Tucker-Lewis index; CFI: comparative fit index.
